# Interventions to increase adherence to micronutrient supplementation during pregnancy: a systematic review

**DOI:** 10.1111/nyas.14545

**Published:** 2021-01-05

**Authors:** Filomena Gomes, Shannon E. King, Diana Dallmann, Jenna Golan, Ana Carolina Feldenheimer da Silva, Kristen M. Hurley, Gilles Bergeron, Megan W. Bourassa, Saurabh Mehta

**Affiliations:** 1 The New York Academy of Sciences, New York City, New York.; 2 Human Nutrition, Johns Hopkins Bloomberg School of Public Health, Baltimore, Maryland.; 3 School of Human Nutrition, McGill University, Montreal, Quebec, Canada.; 4 Division of Nutritional Sciences, Cornell University, Ithaca, New York.; 5 Department of Nutrition in Public Health, University of State of Rio de Janeiro, Rio de Janeiro, Brazil.; 6 Vitamin Angels, Santa Barbara, California

**Keywords:** pregnancy, micronutrients, supplementation, adherence, compliance

## Abstract

Prenatal micronutrient supplements are cost-effective in reducing nutritional deficiencies and adverse pregnancy and birth outcomes. However, poor adherence remains a potential barrier to the successful implementation of these supplementation programs. This systematic review assessed the effectiveness of interventions designed to increase adherence to prenatal micronutrient supplementation. Following the Cochrane Collaboration Methodology, literature searches were conducted in six electronic databases and gray literature (on July 24, 2020), and abstract screening, data extraction, and risk of bias assessment were conducted independently by two reviewers. We included 22 studies. Interventions that resulted in increased adherence were most of the education-based strategies, consumption monitoring by volunteer health workers or family members, SMS reminders, free provision of supplements, a multicomponent intervention with community mobilization, and a participatory action research intervention. In several studies, increased adherence was accompanied by beneficial effects on pregnancy and birth outcomes. Given the heterogeneity of study designs and methods used to define and measure adherence, a meta-analysis was not appropriate. We identified several potentially effective strategies to improve supplementation adherence, which may need to be adapted to specific contexts when considered for program implementation. However, additional high-quality studies are critically needed to effectively guide policies and programs.

## Introduction

The first 1000 days of life, starting at conception and continuing to 2 years of age, are a critical window for growth and development.^1^ Pregnancy requires an adequate intake of key micronutrients (such as vitamins A, D, E, B_6_, folic acid, B_12_, and C, and the minerals iron, zinc, iodine, copper, and selenium) to accommodate maternal and fetal demands. Many of these micronutrients are required in increased doses, some by as much as 50%, during this critical stage of life.^2,3^ Micronutrient deficiencies during pregnancy have immediate and long-term detrimental effects, such as maternal mortality, pregnancy loss, congenital disorders, low birth weight, mortality in infancy, stunting, as well as increased risk of impaired cognitive development and cardiometabolic risk later in life.^2,4,5^ Thus, prenatal micronutrient supplementation is recommended to address these vitamin and mineral deficiencies and to reduce the risk of adverse pregnancy and birth outcomes. Single-nutrient interventions that have been shown to be effective in improving maternal and child outcomes include supplementation with iron to reduce iron deficiency anemia and low birth weight, preconceptional folic acid to prevent neural tube defects, iodine to prevent congenital iodine deficiency syndrome, zinc to reduce the risk of preterm birth, and calcium to reduce preeclampsia in populations with low calcium consumption.^2,3^ Recent systematic reviews of randomized trials have also shown that multiple micronutrient supplements, containing 15 vitamins and minerals designed to address the varying micronutrient needs of pregnant women, result in decreased risks of stillbirth, low birth weight, preterm birth, and being born small-for-gestational age,^6,7^ and are a cost-effective intervention when compared with iron and folic acid (IFA) supplements.^4,8^

Despite the availability and demonstrated efficacy of prenatal micronutrient supplements, the implementation of such interventions continues to be challenging—a special concern since micronutrient deficiencies during pregnancy remain highly prevalent, particularly in low- and middle-income countries (LMICs). For instance, the 2019 Global Nutrition Report shows that anemia still affects 40% of pregnant women worldwide, and none of the 194 evaluated countries was on track to meet the 2025 Global Nutrition Target of reducing anemia by 50% in women of reproductive age.^9^ Poor adherence is one of the main barriers to a successful micronutrient supplementation program, even in settings with high coverage rates (i.e., when a large proportion of pregnant women receive the supplements). An analysis of Demographic and Health Surveys from 22 LMICs showed that while 83% of pregnant women had at least one antenatal care visit and 81% received IFA supplements during that visit, only 8% adhered to the recommended dose (defined as at least 180 tablets during the entire pregnancy).^10^ Another large-scale survey conducted in China showed similar results, even though it used a lower cutoff to define adherence (i.e., 90 tablets), with adherence varying by micronutrient, from 0.6% for iron up to only 11.7% for calcium supplements.^11^

Adherence has been defined as “the extent to which a patient’s behavior matches the agreed recommendations from a healthcare provider.”^12^ Many behavior theories exist to explain the constructs related to adopting positive healthy behavior. In particular, within the literature that has examined adherence to micronutrient supplements in pregnancy, awareness and knowledge have been considered influential constructs for behavior adoption.^13^ Furthermore, other factors, such as age, education level, unplanned pregnancy, lack of time, forgetfulness, high cost, side effects, or difficulty in taking tablets, are also known to influence adherence.^14–16^ Understanding which strategies will lead pregnant women to increased prenatal micronutrient supplement consumption will help maximize the potential benefit of this intervention.^17^ Examples of such strategies could include training healthcare professionals, delivering individual counseling or group educational sessions to pregnant women, sending reminders through text messages, providing financial incentives, and providing family and peer support. This research question was, to some extent, addressed in a previous systematic review of studies designed to increase awareness, knowledge, and consumption of folic acid before and during pregnancy.^13^ However, this review published in 2008 focused only on folic acid, only included studies published between 1992 and 2005, and most interventions were delivered at the population level (without a control group). By contrast, our study aimed to systematically assess and synthesize all existing evidence about targeted interventions designed to increase adherence to any micronutrient supplementation during pregnancy.

## Methods

This systematic review followed the Cochrane Collaboration Methodology^18^ and the Preferred Reporting Items for Systematic Reviews and Meta-analyses (PRISMA) reporting guidelines,^19^ as described in a previously published protocol.^20^

The protocol has also been registered on the International Prospective Register of Systematic Reviews (PROSPERO), the University of York Centre for Reviews and Dissemination (https://www.crd.york.ac.uk/prospero/; registration number CRD42019146814).

### Criteria for considering studies for this review

#### Types of studies.

The study designs included in this review were randomized controlled trials and nonrandomized studies that included a comparison group. Studies without a comparison group were excluded.

#### Types of participants.

The target population was pregnant women who were taking any micronutrient supplements in the context of antenatal care. There was no limit on the length of gestation at the time of enrollment in the study nor on the type of setting (from low- to high-income countries; urban and rural areas). Studies conducted in institutionalized pregnant women were excluded because of the influence that institutionalization can have on adherence.

#### Types of interventions.

We included studies that used targeted interventions designed to improve adherence (i.e., intake) to the recommended prenatal micronutrient supplementation regimen, such as family support, education, or counseling. We considered studies using any micronutrient or combination of micronutrients provided as a powder, liquid (e.g., syrups and suspensions), or pill/tablet, for any duration and frequency; however, the supplementation regimen (i.e., nutrient content and duration) provided in the different groups of the study had to be the same. We included studies that compared one targeted micronutrient adherence intervention to either (1) usual care or no intervention (i.e., no intervention aimed at improving adherence to micronutrient supplements) or (2) another targeted micronutrient adherence intervention. Interventions delivered at the population level (i.e., with no comparison group, such as mass media campaigns) or using fortified or enriched foods were excluded.

#### Types of outcome measures.

The primary outcomes were adherence to micronutrient supplements, as defined by the study authors, and adverse gastrointestinal symptoms (nausea, vomiting, and diarrhea). Secondary outcomes were other adverse effects and pregnancy and birth outcomes, such as levels of hemoglobin and rates of anemia or low birth weight.

### Search methods for identification of studies

The literature searches were conducted in six electronic bibliographic databases (MEDLINE (via PubMed), Embase, Scopus, Web of Science, Scielo, and Cochrane Library), as well as in the gray literature (WHO Library), from inception to July 24, 2020. There were no language or date restrictions. An example of a search strategy used in MEDLINE (via Pubmed) is provided in the Supplementary text (online only).

Study duplicates were removed initially in the bibliographic software Zotero (version 5.0.75), where all the results were merged, followed by further deduplication in the Covidence systematic review software.^21^

### Data collection and analysis

#### Selection of studies.

All the retrieved results were screened against the eligibility criteria by two reviewers (i.e., two of the authors: D.D., J.G., S.K., A.S., and F.G.) independently, using Covidence. In the case of disagreement, a third independent reviewer (M.W.B.) resolved the conflict. Similarly, the full text of the potentially eligible studies was independently assessed by two review team members to determine whether they met the inclusion criteria. When necessary, study authors were contacted to obtain additional information and the reasons for exclusion were documented.

#### Data extraction and management.

Data extraction was conducted independently by two reviewers (i.e., two of the authors: D.D., J.G., S.K., A.S., and F.G.), using a standardized data extraction template in Covidence, and a third independent reviewer identified and resolved discrepancies. Extracted data included study design, study setting (urban, rural, or mixed), classification of country by income (as per the 2020 World Bank classification:^22^ low-income, lower-middle-income, upper-middle-income, or high-income economies), baseline characteristics related to the study population and participant demographics, description of the provided micronutrient supplement, description of the intervention used to increase adherence and comparison group(s), and details of the relevant outcomes (including definition and method used to measure adherence). Multiple attempts to contact study authors were made to obtain missing data or clarify questions related to the methodology of the study.

#### Assessment of risk of bias in included studies.

The risk of bias assessment was conducted by two review authors independently, and disagreements were resolved by a third independent reviewer. The study design determined the tool used to assess risk of bias.

The risk of bias for randomized controlled trials was assessed with the 2011 Cochrane Collaboration’s tool for assessing risk of bias using the following criteria: random sequence generation; allocation concealment; blinding of participants, personnel, and outcomes (assessed separately for adherence and pregnancy and birth outcomes); incomplete outcome data (assessed separately for adherence and pregnancy and birth outcomes); selective outcome reporting; and other sources of bias.^18,23^ Results were categorized as low, unclear, or high risk of bias.

The risk of bias for nonrandomized studies was assessed with the “ROBINS-I” (Risk Of Bias in Non-randomised Studies of Interventions) tool.^24^ This tool requires the assessment of seven risk-of-bias domains, including bias due to confounding; bias in the selection of participants into the study; bias in the classification of interventions; bias due to deviations from intended interventions; bias due to missing data (assessed separately for adherence and pregnancy and birth outcomes); bias in the measurement of outcomes (assessed separately for adherence and pregnancy and birth outcomes); and bias in the selection of the reported results. The results of the judgment for each domain and for the final overall bias were categorized as low, moderate, serious, or critical risk of bias, or no information.

#### Data synthesis and analysis.

Given the heterogeneity of study designs of the included studies and the variable methodologies used to report and measure adherence, it was not possible to perform a meta-analysis. Instead, a narrative analysis of the included studies was conducted, with a synthesis of all the interventions used and a description of the effects of these interventions.

### Methodological amendments to the protocol

Subgroup and sensitivity analyses were initially planned as per the protocol,^20^ but the inability to conduct a meta-analysis rendered this impossible.

## Results

### Results of the search

**Figure 1.**
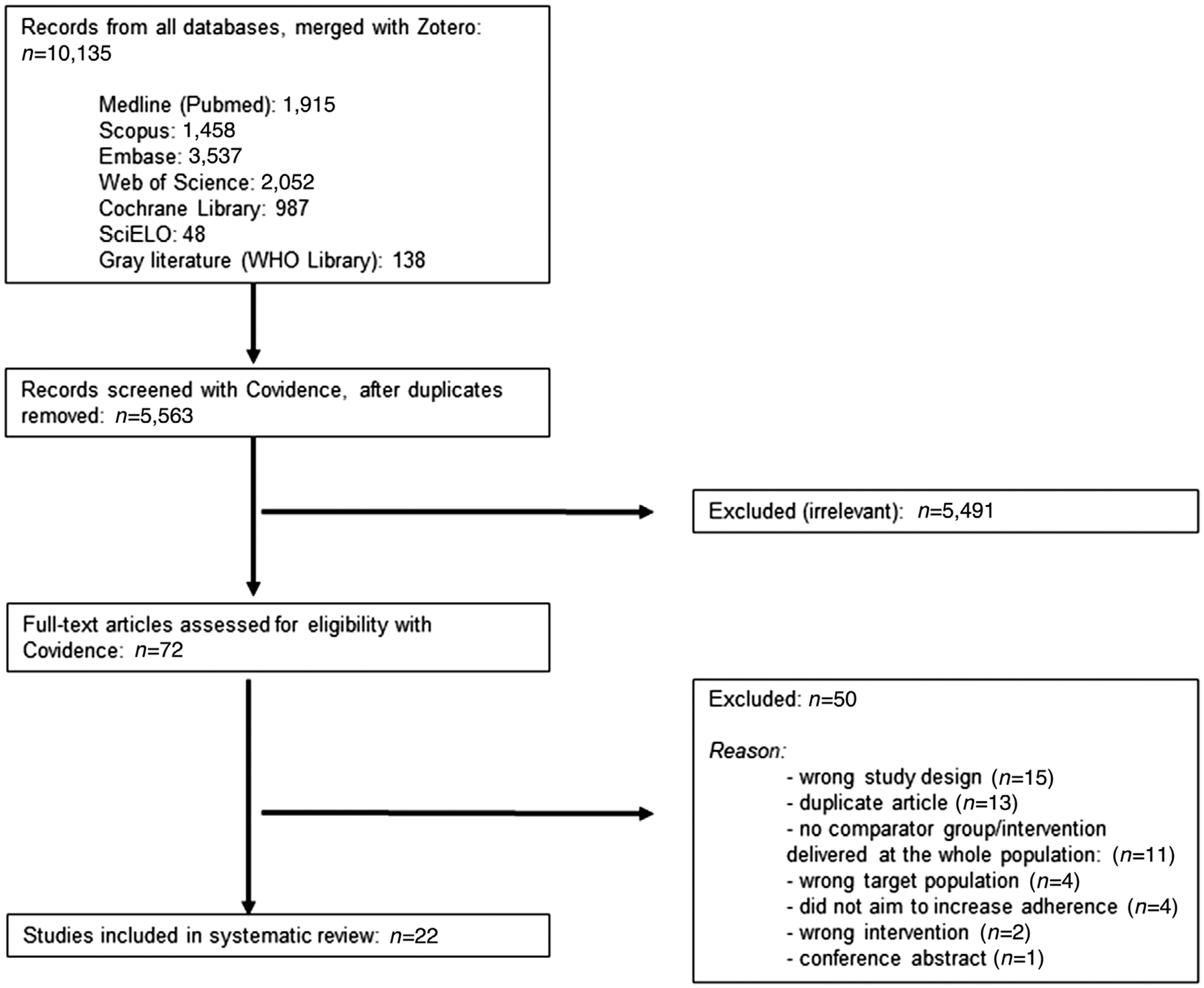
PRISMA flow diagram of literature search and selection process.

A total of 10,135 abstracts were retrieved from the literature searches (Fig. 1). After discarding duplicates, 5563 abstracts were screened in duplicate for eligibility. Of the 72 full-text articles that were assessed for eligibility, 50 were excluded for the following reasons: duplicate articles, conference abstracts, intervention delivered at the population level (lack of the comparator group), and ineligible study design or intervention or target population. Ultimately, 22 studies were included in this review.

### Description of included studies

Of the 22 studies included in this systematic review, 14 were randomized controlled trials (RCTs) (including four cluster-randomized trials) and eight were nonrandomized studies. Studies were published between 2009 and 2020, and all of them used a pre- and posttest design, except for one study,^25^ which used a posttest design only. Table 1 provides an overview of the characteristics of the included studies.

All studies were conducted in countries with lower-middle or upper-middle income economies. Over half of the studies were conducted in Asia (Nepal,^26^ India,^27–33^ Bangladesh,^34^ Indonesia,^25,35–39^ and Thailand^40^), with only two studies conducted in Africa (Senegal^41^ and Kenya^42^) and four conducted in the Middle East (Jordan^43^ and Iran^44–46^).

Of these 22 studies, 10 used an IFA supplement (containing 60–100 mg of iron and 250–500 μg of folic acid), nine used an iron supplement (containing 30–60 mg of iron), one utilized a folic acid supplement (dose not specified), one utilized a combination of iron, folic acid, and calcium supplements (containing 60 mg of iron, 400 μg of folic acid, and 500 mg of calcium), and one used an iodine-supplemented multivitamin (dose not specified). The majority of the studies (*n* = 20) had two groups, either comparing one intervention with one control group or one intervention with another intervention; however, two studies had three^36^ and four groups,^26^ comparing a control group with multiple intervention arms (Table 1).

Collectively, the studies covered a wide range of targeted micronutrient adherence interventions, including education-based ones, education with consumption monitoring, consumption monitoring alone, participatory action research, SMS reminders, free provision (versus purchase) of supplements, different forms of the supplements (i.e., capsule versus tablet), and multicomponent interventions (e.g., education with community mobilization).

Study sample sizes ranged from 60 to 4615 participants; however, two-thirds of the studies had fewer than 200 participants. While all studies were conducted on pregnant women, some specified the inclusion criteria for women within a certain trimester of pregnancy, while others did not provide information on the gestational age of the participants at the time they were enrolled or received the intervention. Five studies were conducted in anemic pregnant women.^29,30,37,39,43^

As per our inclusion criteria, adherence was assessed in all studies (Tables 2 and 3). Nevertheless, the method used to measure adherence differed significantly between the studies, including prevalidated scales (e.g., Morisky Medication Adherence Scale-8 (MMAS-8) questionnaire), self-reported recall, supplement compliance documentation cards, direct observation, and pill counting (Tables 2 and 3). In addition, the definition of adherence also varied significantly between studies. Definitions included a designated cutoff for supplement intake to dichotomize those who were adherent and non-adherent (e.g., at least 80% or 100 supplements taken), continuous measurements of the number of supplements consumed (e.g., a total number of tablets taken in 3 months), and Likert scale results (e.g., a behavior scale that ranged from 1 to 5). The studies utilizing a cutoff point to dichotomize results varied from consumption of 70% to 100% of the expected supplement dose, and from 75 to 180 tablets consumed (Tables 2 and 3). It should also be noted that, although almost all studies had a pre- and posttest design (which allows them to show the mean differences of adherence measures between baseline and endline), 12 studies reported measures of adherence for the posttest only. Because of all these methodological differences, it was not possible to directly compare the effect of the intervention on adherence between studies.

Adverse gastrointestinal symptoms and the secondary outcomes of interest were reported by fewer studies. Adverse effects were only reported in four studies;^28,35,37,42^ hemoglobin levels were assessed in 13 studies;^26,28–32,35,36,38,39,41,43,46^ serum ferritin was assessed in five studies;^28,30,32,41,44^ hematocrit was assessed in two studies;^39,46^ and only one study each assessed plasma folate,^45^ serum transferrin,^28^ erythrocyte protoporphyrin concentration,^41^ total iron binding capacity,^30^ urinary iodine concentration,^40^ and low birth weight^39^ (Tables 2 and 3).

### Effects of interventions

Sixteen of the 22 studies (73%) found a significant between-groups difference (comparing control to intervention or multiple intervention groups) in the adherence to the prescribed supplementation regimen (Tables 2 and 3). Below is a summary of the effect of the interventions provided by type of interventions.

#### Education-based interventions (10 studies).

Nine of the 10 education-based interventions resulted in a significant increase of adherence to supplementation. Surtimanah *et al.*^25^ found that women who received individual counseling from midwives during antenatal care had a significantly higher mean intake of iron tablets consumed in comparison with women who attended group classes for pregnant women. Vernissa *et al.*^37^ demonstrated that both the group that received individual counseling by a pharmacist at the health center and the group that received education leaflets at the health center had a significant increase of adherence (from baseline to endline), but there was no statistically significant difference between groups. Prinja *et al.*^27^ found that using an mHealth application^a^ as a job aid for community health volunteers to provide custom counseling messages resulted in a significant difference-in-difference (12.7%) on adherence rates compared with the group that received standard antenatal care. Abujilban *et al.*,^43^ Heryadi *et al.*,^38^ and Saha *et al.*^30^ used education-based strategies (i.e., a health information package, pharmacist counseling using a leaflet, and targeted counseling outside of antenatal care, respectively) that resulted in statistically significant increases in supplementation adherence when compared with the control groups that received standard antenatal care. Jalambadani *et al.*^44^ compared the use of education sessions (intervention group) with the provision of education pamphlets (control group) and observed a significant increase on a behavior scale in the intervention group. Two years later, the same authors (Jalambadani *et al.*^45^) published another study that found significant differences in the mean scores of consumption of folic acid supplements between those who received the education sessions (intervention group) and those receiving antenatal care (control group). The intervention provided in the study of Nahrisah *et al.*^39^ included two home visits from a trained midwife (with individual education through pictorial handbooks and counseling), which resulted in a significantly higher intake of supplements when compared with standard antenatal care (control group). In contrast to these effective interventions, Anitasari and Andrajati^35^ observed that the changes in adherence were not statistically significant between the two groups.

Within these education-based interventions, adverse side effects were measured by two studies, which observed no significant differences in this outcome between the groups.^37,47^ The most common side effects reported by Anitasari and Andrajati^35^ were nausea/vomiting and constipation. Five studies assessed hemoglobin levels, four of which observed significant increases of hemoglobin between intervention(s) and control groups,^30,38,39,43^ whereas one study found no significant differences in hemoglobin changes between both intervention groups.^35^ Ferritin was assessed in two studies and both reported significant differences between the intervention and control groups, with higher levels observed in the intervention group.^30,44^ One study reported a significant increase in the intervention group’s plasma folate levels, as opposed to the control group.^45^ Only one study assessed birth weight as an outcome, which was statistically significantly higher in the intervention group (versus control).^39^ This effect size was also clinically meaningful, as the adjusted mean birth weight of the intervention group was 3324 g, in comparison with the adjusted mean birth weight of control group, which was 2975 grams. The intervention of this study consisted of two home visits (45–60 min) from a trained midwife, with individual education and counseling.

#### Education and consumption monitoring (two studies).

The two studies that used education in conjunction with consumption monitoring strategies employed multiple intervention groups and found significant differences in adherence between study groups. The study from Darmayanti *et al.*^36^ showed that the use of consumption monitoring cards (in addition to counseling/educational leaflets) resulted in higher adherence than the use of counseling/educational leaflets alone, and there were significant differences between the changes of hemoglobin in all three study groups. Adhikari *et al.*^26^ showed a higher adherence in the group that received education and a pill count when compared with the group with a pill count assessment alone. Compared with the control group (standard ANC), there were significant improvements in hemoglobin changes and anemia prevalence in the group that received education with pill counting, but not in the group with pill counting alone.

#### Consumption monitoring (two studies).

Consumption monitoring strategies, that is, supervised consumption of supplements by volunteer health workers or family members, proved to be effective in improving adherence to supplementation in both trials that used this intervention: Ahamed *et al.*^28^ and Balakrishnan *et al.*^29^ While both studies found significant differences between groups in hemoglobin levels from baseline to endline, there was no difference in changes of anemia rates in the study that assessed this outcome.^28^

#### Participatory action research interventions (two studies).

Two studies used a participatory action research approach. One of them (Chaiopanont and Taneepanichsakul) aimed at improving iodine consumption following a four-step participatory action research design that implemented screening, provision, counseling, and home visits, and found no significant differences in adherence to the iodine-supplemented multivitamins.^40^ However, they observed significant differences in urinary iodine concentrations between the intervention and control groups at the endline (which could be explained by other variables such as the use of more iodized salt, iodine-supplemented fish sauce, or iodine-rich foods). The other study (Shivalli *et al.*) used a trial of improved practice design to increase the consumption of IFA supplements (with three visits for assessment; negotiation to support behavior change, where pregnant women were asked to select and try new recommended practices; and evaluation). The authors described large differences in the proportion of individuals defined as compliant (i.e., 85% in the intervention group and 38% in the control group); however, statistical significance was not reported.^31^ This intervention resulted in significantly higher hemoglobin levels and greater reductions in the prevalence of anemia when compared with the control group (standard antenatal care).

#### SMS reminders (one study).

Khorshid *et al.*^46^ assessed the effect of SMS reminders and educational health messages, which resulted in a significantly higher adherence and prevalence of “high compliance” compared with standard antenatal care. Despite these positive effects on adherence, no significant differences were observed in hemoglobin or hematocrit levels between both groups.

#### Free versus purchased supplements (one study).

Seck *et al.*^41^ compared the provision of free supplements at antenatal care (intervention group) to the receipt of the prescription that required filling and paying of supplements at a pharmacy (control group), and they observed significantly higher consumption of supplements among those receiving the free supplements. Similarly, there were statistically significant differences in the mean levels of hemoglobin, erythrocyte protoporphyrin, and serum ferritin at follow-up between the control and the intervention groups.

#### Capsule versus tablet supplements (one study)

Srivastava *et al.*^32^ compared the provision of iron tablets (control group) with iron capsules (intervention group) and observed no significant differences in reported adherence, hemoglobin levels, or ferritin levels between both groups.

#### Multicomponent interventions (three studies).

Three studies used a combination of several interventions. Nguyen *et al.*^34^ assessed the effect of a multicomponent intervention that included community-based promotion events and husband education (among others), which resulted in increased adherence to both IFA and calcium supplements compared with the control group. By contrast, Kamau *et al.*^42^ used an intervention with a community-based distribution of IFA supplements by community health workers, counseling, and weekly follow up with pregnant women in their homes and did not observe significant differences in adherence between the intervention and the control group (receiving standard antenatal care). Nonetheless, the percentage of adverse side effects reported at follow up was generally much lower in the intervention group, which is probably a result of the counseling about managing and mitigating the common side effects of IFA supplements. A similar lack of effect on adherence was observed in the study from Hazra *et al.*,^33^ although when the analysis was confined to the subgroup of the most marginalized women (based on women’s education, caste, and household wealth index), the intervention proved to be effective in increasing adherence, with a statistically significant difference-in-difference between intervention and control. This intervention used self-help groups focused on microfinance activities, with an additional maternal and neonatal health package composed of health discussion and videos at the self-help meetings, distribution of leaflets and letters with information on maternal and child health, community-based meetings to disseminate health messages, support to attend health days, monthly celebrations, and additional meetings for pregnant women.

### Risk of bias

The overall risk of bias was moderate, serious, or critical for all the included nonrandomized studies (Table 4). For the included RCTs, a few studies had low risk of bias through most of the domains (e.g., Adhikari *et al.*,^26^ Srivastava *et al.*,^32^ and Nahrisah *et al.*^39^); 4 out of 14 studies had unclear risk of bias^30,31,38,45^ and none were at high risk of bias for sequence generation; 9 out of 14 studies had unclear^29–31,38,44–46^ or high^28,41^ risk of bias for allocation concealment; and 11 out of 14 studies had unclear^30,34,38,39,41,43–46^ or high^29,31^ risk of bias for blinding of outcome assessors for adherence (Table 5).

## Discussion

Defining and quantifying adherence, as well as providing an estimate of the effect size of an intervention on adherence for a pooled body of evidence, is challenging. Previously published systematic reviews used different cutoffs to define adherence to micronutrient supplements in pregnancy, which varied from 70%^48^ to 95%^7^ of the recommended daily supplementation dose or was defined as the intake of micronutrient tablets for 90 days or more.^49^ Given this variability in the definition of adherence and the criticism of using a specific threshold (which could not be proved to be linked to clinical outcomes^50^), in the present work, we decided to report adherence to the micronutrient supplementation regimen as measured and defined by the study authors. Consequently, we found that the included studies used a wide range of methods to measure adherence (from prevalidated questionnaires to self-reported recall or pill counting) and to define adherence (from a minimum number of tablets taken over a certain period of time to the use of behavior scales). In addition, adherence was assessed at different time points, from 1 to 6 months after initiation of the intervention. Moreover, the included studies had different types of study designs (RCTs versus nonrandomized studies), evaluation designs (pre- and posttest versus posttest only), and types of micronutrient supplements provided (with a prominent use of IFA supplements as per recommendations from global guidance^51^). Given this heterogeneity between the studies, a statistical meta-analysis was not feasible nor appropriate for our systematic review.

We found that a variety of interventions were effective in increasing prenatal supplement adherence. Most of these interventions were education-based strategies (used alone or in combination with consumption monitoring), including individual counseling (from midwives at ANC, pharmacists at the health center, or community health workers at home), through education sessions, pictorial handbooks, leaflets, and videos, among others. The use of other interventions based on consumption monitoring by volunteer health workers or family members, SMS reminders, free provision of supplements, a multicomponent intervention with community mobilization, and a participatory action research intervention (with three visits for assessment, negotiation to support behavior change, and evaluation), despite being less prevalent, also effectively increased supplement adherence. Interventions that did not result in increased adherence included the provision of a capsule versus tablet, one participatory action research intervention, and two multicomponent interventions. The types of interventions identified in the present review are in line with the results of a Cochrane review of interventions for enhancing medication adherence,^52^ which included complex interventions with several components, such as intense education and tailored ongoing counseling delivered by allied health professionals/pharmacists, daily treatment support, and sometimes additional support from family or peers. Nevertheless, only a few of those interventions for improving adherence with long-term medication prescriptions improved both adherence and clinical outcome.

Our systematic review shows that most of the interventions that resulted in increased micronutrient supplement adherence also resulted in beneficial effects on pregnancy and birth outcomes (when reported by the study authors), but there were some exceptions. For example, the study from Khorshid *et al.*^46^ showed that, when compared with standard ANC, the use of SMS reminders and educational health messages over 12 weeks resulted in higher adherence to iron supplements but no significant differences were observed in hemoglobin or hematocrit levels between both groups. It is possible that interventions need to be delivered for a longer period of time to have an effect on clinical outcomes, and there may be other factors influencing the response of the blood parameters of anemia to the iron supplements. In addition, the difference between the mean number of tablets consumed in each group (i.e., 80.5 tablets in the intervention group versus 67.2 tablets in the control group) may not be large enough to result in significant differences in hemoglobin or hematocrit levels between both groups.

While we considered studies using any type of micronutrient supplement, as mentioned above, most of included studies used iron or IFA supplements. For the studies that reported doses of these micronutrients, iron was provided in high amounts (60–100 mg of elemental iron), except for one study that used 30 mg of iron.^46^ The dose of iron that is typically provided in multiple micronutrient supplements is lower, that is, 30 mg. It is unclear whether studies that use supplements containing high doses of iron present inherently lower levels of adherence (as a consequence of more side effects) than studies that use supplements with lower doses of iron, such as multiple micronutrient supplements.

While conducting the literature searches, we found some ongoing studies that could not be included at this stage but may be included in a future update of this systematic review, such as: (1) an RCT assessing whether a social norm–based intervention can increase the uptake of iron folic acid supplements and iron-rich foods to reduce anemia in Indian pregnant women;^53^ and (2) a cluster RCT assessing the effects of a video-based health education package provided to Ethiopian pregnant and lactating women on the knowledge, attitude, and practice of recommended health, including adherence to IFA supplementation.^54^

In addition to these ongoing studies, it should be noted that the 22 studies included in this systematic review were published over a span of 11 years, reflecting the urgent need to tackle this problem of suboptimal intake of micronutrient supplements during pregnancy, particularly in low- and middle-income settings.

There are a number of limitations in the present work. First, most studies used self-reported measures of adherence, which may be subject to several biases (e.g., recall bias and response bias). Second, we included a few studies that reported adherence as an outcome but did not report data on other pregnancy and birth outcomes. Thus, we do not know whether these interventions that increased adherence also resulted in more objective and clinically relevant outcomes for both the pregnant mother and her child. Nonetheless, it should be noted that a lack of clinical outcomes in implementation research is not necessarily a limitation, since such studies aim to test interventions for which the evidence of efficacy already exists and rarely can (or should) be designed to determine the assessment of clinically responsive outcomes. Third, we found that some studies had an overall low quality, as reflected in the risk of bias assessment, with serious methodological limitations (particularly among the nonrandomized studies) and relatively small sample sizes. Thus, their findings need to be interpreted with caution. Fourth, none of the included studies were conducted in low- or high-income countries, which limits the extrapolation of the evidence to these settings. A previous systematic review of interventions designed to increase knowledge, awareness, and consumption of folic acid in women of reproductive age^13^ found that the included studies were conducted in the United States, Australia, Europe, and Israel. The predominant use of mass media channels of communication, such as TV and internet, in the interventions of that systematic review conducted in 2008 suggests that low- and middle- and high-income settings might need different interventions. On the other hand, advocacy capabilities and access to mobile communications in low- and middle-income settings have changed dramatically in the past decade such that some of the adherence interventions tested in the past (e.g., use of education leaflets) may not be used in the future, and there is a trend for increased use of remote counseling services.

The strengths of this study include the fact that there was no language restriction (which led to the inclusion of one study written in Indonesian^37^) and that we did multiple attempts to contact study authors, which resulted in the inclusion of important (missing) data that had not been published. In addition, we followed the rigorous Cochrane methodological requirements and conducted the literature search in a large number of databases (*n* = 7, including gray literature).

To our knowledge, this is the first systematic review to assess the existing literature to determine the effectiveness of interventions designed to increase adherence to micronutrient supplements in pregnancy, following the Cochrane methodology.

Current evidence suggests that a number of strategies play a role in increasing adherence to the recommended prenatal micronutrient supplementation regimen, which may need to be adapted to specific contexts (e.g., country, region, or culture). Additional high-quality and adequately powered studies, using feasible and sustained interventions and objective adherence measures, are warranted to determine other efficacious strategies that maximize the benefit of micronutrient supplements during this critical stage of life. While it is not reasonable to recommend the use of a specific cutoff to define adherence based on the results of this systematic review, we encourage all authors of future studies assessing adherence to micronutrient supplementation to specify the recommended dosage and report the “number of supplements consumed divided by the expected number of pills to be taken.” This assessment takes into consideration the beginning of the supplementation period and allows a better comparison of adherence between different studies.

## Supplementary Material

Supplementary Material

## Figures and Tables

**Table 1.** Overview of the characteristics of the included studies

Study author, year	Country (income^a^); study setting, when specified	Supplement (tablets or capsules, and dose when specified)	Control Group (C)	Intervention 1 (I1)	Intervention(s) 2 and 3 (I2 and I3)	Total sample size	Study design; evaluation design	Study participants	Age, mean ± SD or % per age group
**Nonrandomized studies**									
Anitasari (2017)^35^	Indonesia (UM)	Iron tablets		I1: Health education leaflets	I2: Health education through short message service (SMS) reminders	74	Quasi-experimental; pre- and posttest design	Pregnant women in second or third trimester	<20 y: I1: 0%, I2: 2.8% 20–25 y: I1: 76.3%, I2: 66.7% >35 y: I1: 23.7%, I2: 30.5%
Chaiopanont (2019)^40^	Thailand (UM)	Iodine supplemented multivitamins	Standard ANC	Participatory Action Research. Interventions included screening iodine status, provision supplements, counseling and home visits		88	Participatory action research; pre- and posttest design	Pregnant woman	I1: 23.47 y ± 5.4 C: 26.83 y ± 6.25
Darmayanti (2019)^36^	Indonesia (UM); urban	Iron and folic acid tablets (>60 mg iron and 400 mg folic acid)	Counseling and educational leaflets	I1: Counselling and educational leaflets with government issues monitoring card	I2: Counseling and educational leaflets with research monitoring card	120	Quasi-experimental; pre- and posttest design	Pregnant women	
Hazra (2019)^33^	India (LM); rural	Iron and folic acid tablets	Self-help groups focused on microfinance activities	Self-help groups focused on microfinance activities with an additional mater nal and neonatal health package, including: health discussion and videos at the self-help meetings, distribution of leaflets and letters with information on maternal and child health, community-based meeting to disseminate health messages, etc.		Baseline: 4615 Endline: 4250	Quasi-experimental; pre- and posttest design	Women who had given birth in the past 12 months	Baseline I: 26.2 y ± 4.48 C: 26.2 y ±4.47 Endline I: 25.9 y ± 4.66 C: 25.8 y ±4.73
Kamau (2020)^42^	Kenya (LM)	Iron and folic acid supplements	Standard of care (ANC)	Community-based distribution of supplements by community health workers, counseling and weekly home follow-ups of pregnant women		340	Quasi-experimental; pre- and posttest design	Pregnant women (<33 weeks pregnancy)	I: 25.7 y ± 5.7 C: 25.6 y ± 5.9
Prinja (2017)^27^	India (LM); rural	Iron and folic acid supplements	Standard ANC	mHealth application job aid for health volunteers to provide customized counseling messages		Matched sample: 633	Quasi-experimental; pre- and posttest design	Women with children between age 29d and 6m	
Surtimanah (2019)^25^	Indonesia (UM)	Iron tablets		Pregnant women group classes	Individual counseling provided by midwives at ANC	60	Quasi-experimental; pre- and posttest design	Pregnant women	
Vernissa (2017)^37^	Indonesia (UM)	Iron tablets	Counseling by pharmacist at the health center	Leaflet given to women at the health center		158	Quasi-experimental; pre- and posttest design	Pregnant women with anemia	< 20 y: C: 8.9%, I: 13.9% 20–35 y: C: 77.2%, I: 69.7% >35 y: C: 13.9%, I: 16.4%
**Randomized controlled studies**									
Abujilban (2019)^43^	Jordan (UM)	Iron supplements	Standard ANC, including provision of iron supplements	Health Information Package Program (health education videos, individualized teaching and video promotion via mobile application)		200	Randomized controlled trial; pre- and posttest design	Pregnant women with anemia, second trimester	C: 29.6 y I: 28.4 y
Adhikari (2009)^26^	Nepal (LM); rural	Iron tablets (60 mg of ferrous sulphate)	Standard ANC	Pill count assessment	Intervention 2: targeted counseling session Intervention 3: targeted counseling session and pill count	284	Randomized factorial control trial; pre- and posttest design	Pregnant women, second trimester	C: 25.4 y ± 5.1 I1: 27 y ± 5.5 I2: 27.3 y ± 6.3 I3: 27.4 y ± 6.4
Ahamed (2018)^28^	India (LM); rural	Iron (100 mg) and folic acid (500 mg) tablets (either 1 or 2 depending on baseline hemoglobin)	Iron and folic acid tablet provision	Iron and folic acid tablet provision and supervised consumption by volunteer health workers		368	Randomized controlled trial; pre- and posttest design	Pregnant women, first trimester	C: 22.9 y ± 2.6 I: 23 y ± 2.8
Balakrishnan (2019)^29^	India (LM); rural	Iron tablets (ferrous ascorbate)	Standard ANC, including provision of iron tablets	Direct observation of supplement consumption by a family member		140	Randomized controlled trial; pre- and posttest design	Pregnant women, second trimester, and mild and moderate anemia	18–20 y: 52.9% 21–25 y: 42.9%
Heryadi (2017)^38^	Indonesia (UM)	Iron tablets (60 mg, ferrous fumarate)	Standard ANC	Pharmacist counselling using leaflet		192	Randomized controlled trial; pre- and posttest design	Pregnant women	21–35 y: C: 83.3%, I: 78.1% <21 y or >35 y: C: 16.7%, I: 21.9%
Jalambadani (2018)^44^	Iran (UM); urban	Iron supplements	Education pamphlet	Educational sessions at the health center		160	Randomized controlled trial; pre- and posttest design	Pregnant women, second and third trimester	I: 10.53 y ± 25.75 C: 10.77 y ± 26.35^b^
Jalambadani (2020)^45^	Iran (UM); urban	Folic acid supplements	Standard ANC	Educational sessions at the health center		180	Quasi-experimental trial, cluster randomization; pre- and posttest design	Pregnant women, first trimester	I: 29.75 y ± 10.53 C: 29.35 y ±10.77
Khorshid (2014)^46^	Iran (UM); urban	Iron tablets (30 mg ferrous sulphate)	Standard ANC	Standard ANC with SMS reminders and educational health messages		93	Randomized controlled trial; pre- and posttest design	Pregnant women, second trimester	I: 24.7 y ± 4.2 C: 25.5 y ± 6.6
Nahrisah (2020)^39^	Indonesia (UM)	Iron and folic acid tablets	Standard of care ANC	Standard of care ANC + two home visits (45–60 min) from a trained midwife, including individual education through pictorial handbooks and individual counselling		140	Quasi-experimental, simple random sampling; pre- and posttest design	Pregnant women with anemia. Mean gestational age: 20 w	I: 28.4 y ± 3.51 C: 28.2 y ± 3.8
Nguyen (2017)^34^	Bangladesh (LM); mixed	Iron (60 mg), folic acid (400 mg), and calcium (500 mg) tablets	Standard ANC care (counseling), provision of iron, folic acid, and calcium supplements	Intensified interpersonal counseling by health workers, home visits, free supplement provision, husband education forums, community-based promotion events, and health worker performance incentive structure		2000	Cluster randomized trial; pre- and posttest design	Recently mothers (children <6 m)	
Saha (2018)^30^	India (LM); rural	Iron and folic acid tablets	Standard ANC	Targeted counseling messages delivered outside of ANC		118	Randomized controlled trial; pre- and posttest design	Pregnant women with anemia, first trimester	I: 22.7 y ± 2.9 C: 22.8 y ± 3.1
Seck (2009)^41^	Senegal (LM); urban	Iron and folic acid tablets (65 mg elemental iron and 250 mg folic acid)	Standard of care: received prescription for iron and folic acid tablets that needed to be filled and paid for	Free provision of iron and folic acid tablets at ANC visit		221	Cluster randomized controlled trial; pre- and posttest design	Pregnant women, second trimester	I: 27.3 y ± 0.6 C: 26.6 y ± 0.6
Shivalli (2015)^31^	India (LM); rural	Iron and folic acid (100 mg elemental iron and 0.5 mg folate) tablets	Standard ANC (solely assessment and evaluation visit)	Trial of Improved Practices (three visits, including assessment visit, negotiation to support behavior change, and evaluation visit)		98	Cluster randomized controlled trial; pre- and posttest design	Pregnant women, second trimester	I: 23.2 y ± 2.8 C: 22.9 y ± 3.5
Srivastava (2019)^32^	India (LM); mixed	Iron and folic acid (100 mg elemental iron and 500 mg folic acid) tablets or capsules	Iron tablets (ferrous sulphate)	Iron capsules (ferrous fumarate)		204	Randomized controlled trial; pre- and posttest design	Pregnant women, second and third trimester	I: 23.8 y ± 3.3 C: 23.5y ± 3.6

a Income by World Bank Classification: LM, l-middle-income economies; UM, upper-middle-income economies.

b Study authors seem to have reported standard deviation (mean), rather than mean (standard deviation).

C, control group; I, intervention group; ANC, antenatal care; N.A., not available.

**Table 2.** Outcome analysis for nonrandomized studies

	Adherence					Pregnancy and birth outcomes
Study author (year)	How it was measured	When it was measured	How it was defined	Results, as reported	Adverse effects, as reported	Hemoglobin (g/dL) and other biomarkers
**Education-based strategies (iron/iron and folic acid supplements)**						
Anitasari (2017)^35^	Self-reported questionnaire	One month after intervention	Average MMAS-8 score^a^	Mean (SD) score: I1 pretest: 4.26 (2.36), I1 posttest: 3.45 (1.94), (*P* = 0.018) I2 pretest: 3.50 (2.36); I2 posttest: 2.94 (2.11), *P* = 0.180 Average change: I1: 0.81; I2: 0.56; *P* = 0.576	Had side effects: I1: 16 (42.1%) I2: 14 (38.9%) *P* = 0.778 Most common: nausea/vomiting and constipation	Mean hemoglobin (SD): I1 pretest: 11.2 (1.36); I1 posttest: 11.1 (1.22), *P* = 0.553 I2 pretest: 11 (1.36), I2 posttest:10.9 (1.19), *P* = 0.789 Average change: I1: −0.05; I2: −0.08, *P* = 0.929
Surtimanah (2019)^25^	Self-reported and tablet compliance card	One month after intervention	Number of iron tablets taken (1 month)	Mean, median (min, max), posttest: I1: 28.87, 30 (21, 30) I2: 29.83, 30 (27, 30), *P* = 0.003		
			Women consuming 30 tablets (1 month)	Prevalence, *n* (%), posttest: I1: 18 (60%); I2: 28 (93.3%)		
Vernissa (2017)^37^	Self-reported questionnaire	One month after intervention	Average MMAS-8 score^a^	Mean (SD) score: I pretest: 3.08 (1.83), I posttest: 1.57 (1.61), *P* < 0.001 C pretest: 2.89 (1.52), C posttest: 1.51 (1.38), *P* < 0.001 Between-groups differences: *P* > 0.05	Experienced side effects, *n* (%): I: 33 (42.8%) C: 29, (36.7%), No side effects, *n* (%): I: 46 (58.2%) C: 50 (63.3%), *P* = 0.515	
Prinja (2017)^27^	Self-reported (cross-sectional survey)	N/A	At least 100 tablets taken	Prevalence (%):^b^ I: pretest 2, posttest 1 C: pretest 14, posttest: 0.4 DID: 12.7%		
**Education and consumption monitoring (iron/iron and folic acid supplements)**						
Darmayanti (2019)^36^	Pill count	90 days after intervention	At least 75 pills taken for 3 months of intervention	Prevalence (%): C posttest: 52.58 I1 posttest: 70.39 I2 posttest: 58.54, *P* = 0.032		Mean hemoglobin difference between baseline and endline: C: reference group I1: −0.5425, I2: −0.6175, *P* < 0.05
**Multicomponent intervention (iron and folic acid supplements)**						
Hazra (2019)^33^	Self-reported administered questionnaire	Cross-sectional survey of women who gave birth within the past 12 months	Consumption of 100 or more tablets during pregnancy	Prevalence (%): I1: pretest 13, posttest 19.1 C: pretest 8.7, posttest 12.9 Adjusted DID: 1.9 (−0.9, 4.8), *P* = 0.186 Prevalence (%) in the most marginalized women: I1: pretest 9, posttest 20.9 C: pretest 3, posttest 7.5 Adjusted DID: 7.3 (0.5, 4.1), *P* = 0.036		
Kamau (2020)^42^	Self-reported	At baseline and endline (before delivery from 36 weeks of gestation)	At least 70% of expected dose in the previous week (5 out of 7 days)	Prevalence (%): I pretest: 63.8, I posttest: 71.4 C pretest: 68.5, C posttest: 74.3. DID: 0.02 (−0.20, 0.24), *P* > 0.05	Percentage of side effects reported was generally much lower in I versus C at the endline	
**Participatory action research (iodine-supplemented multivitamins)**						
Chaiopanont (2019)^40^	Self-reported	9 weeks after intervention	Daily intake of supplement	Prevalence, *n* (%): Pretest: I: 43 (91.5%), C: 38 (92.7%), *P* = 0.836 Posttest: I: 44 (93.6%), C: 36 (87.8%), *P* = 0.344		Concentration of urinary iodine (mg/L), posttest mean (SD); adjusted mean: I: 264.65 (192.50); 386.83 C: 233.03 (158.94); 254.98
			4–6 supplements taken/week	Pretest: I: 4 (8.5%), C: 3 (7.3%), *P* = 0.836 Posttest: I: 3 (6.4%), C: 5 (12.2%), *P* = 0.344		Between-groups difference: *P* = 0.047

Note: When available, outcome results are highlighted in green if differences (within-group and between-groups) are statistically significant, and in red when not statistically significant.

a Morisky Medication Adherence Scale-8 (MMAS-8) questionnaire: a score of 0 indicates complete compliance, 1 and 2 indicate moderate compliance, and >2 indicates noncompliance. A lower MMAS-8 score indicates a higher compliance.

b Prevalence data as reported by the authors (very low numbers).

C, control group; I, intervention group; DID, difference-in-difference ((CI), *P* value).

**Table 3.** Outcome analysis for randomized controlled trials

	Adherence				Pregnancy and birth outcomes, as reported
Study author (year)	How it was measured	When it was measured	How it was defined	Results, as reported	Hemoglobin (Hb) (g/dL), anemia, ferritin (ng/mL) and others
**Education-based strategies (iron/iron and folic acid/folic acid supplements)**					
Abujilban (2019)^43^	Self-reported questionnaire	4 weeks after intervention	Compliance checklist, but no further information provided on what this entailed	Mean (SD), pretest: I: 12.04 (2.44), C: 11.45 (2.39), *P* = 0.09 Mean (SD), adjusted mean (SE), posttest: I: 14.13 (2.68), 13.92 (0.229) C: 11.45 (3.02), 11.66 (0.229), *P* = 0.01	Mean (SD) Hb, pretest: I: 9.66 (0.7), C: 9.55 (0.86), *P* = 0.33 Mean (SD), adjusted mean (SE) Hb, posttest: I: 10.56 (0.96), 10.54 (0.099) C: 9.71 (1.08), 9.723 (0.099), *P* = 0.01
Heryadi (2017)^38^	Pill count	30 days after intervention	Number of pills taken	Prevalence of women consuming: I posttest: 0–15 tablets: 13.5%; 16–30 tablets: 20.8%; 31–45 tablets: 49.0%; 46–60 tablets: 16.7% C posttest: 0–15 tablets: 43.8%; 16–30 tablets: 53.1%; 31–45 tablets: 2.1%; 46–60 tablets: 1% Between-groups difference: *P* < 0.001	Mean (SD) Hb: I pretest: 10.39 (1.24) I posttest: 11.52 (0.92), *P* < 0.001 C pretest: 11.21 (1.24) C posttest: 11.28 (11.53), *P* = 0.300
Jalambadani (2018)^44^	Self-reported questionnaire	6 months after intervention	Three-item behavior scale ranging from highly opposed (score 1) to highly agreeable (score 5)	Mean score (SD):^a^ I pretest: 0.42 (2.25), I posttest: 3.25 (5.45), *P* = 0.013 C pretest: 2.67 (2.75), C posttest: 2.65 (3), *P* = 0.061 Between-groups difference: *P* = 0.002	Mean (SD) ferritin^a^, pretest; posttest: I: 0.93 (11.56); 1.01 (13.61), *P* = 0.01 C: 0.57 (11.61); 0.54 (10.6), *P* = 0.804 Between-groups difference: *P* = 0.010
Jalambadani (2020)^45^	Self-reported questionnaire	3 months after intervention	No further information provided on what the questionnaire entailed	Significant difference between the mean score of consumption of folic acid between I and C (*P* < 0.05); values not provided.	Mean (SD) plasma folate, pretest; posttest: I: 10.25 (0.93); 12.05 (1.03), *P* < 0.01 C: 1.65 (0.67); 10.85 (0.097), *P* = 0.704 Between-groups difference: *P* < 0.01
Nahrisah (2020)^39^	Pill count	15 weeks after intervention	Number of IFA taken	Adjusted mean (SD), posttest: I: 55.5 (18.69); C: 23.2 (11.62), *P* < 0.001	Adjusted mean (SD) Hb, posttest: I: 11.7 (0.37); C: 10.5 (0.5), *P* < 0.001 Adjusted mean (SD) hematocrit (g/dL), posttest: I: 35.2 (0.81), C: 31.2 (0.92), *P* < 0.001 Adjusted mean (SD) birthweight (g), posttest: I: 3324 (354), C: 2975 (279), *P* = 0.006
Saha (2018)^30^	Self-reported	Baseline, midway (second trimester) and endline (third trimester)	Regular intake (no further information on definition)	Prevalence of regular intake, *n* (%): I pretest: 3 (5.1%), I midtest: 19 (32.2%), I posttest: 33 (55.9%) C pretest: 2 (3.4%), C midtest: 3 (5.1%), C posttest: 6 (10.2%) Between-groups difference: *P* = 0.001	Median (SD)^b^ Hb, posttest: I: 10.4 (0.95); C: 9.2 (0.98), *P* = 0.001 Mild anemia (%): I pretest: 37.3%, C pretest: 44.1%. I posttest: 62.7%, C posttest: 40.7% Moderate anemia (n, %): I pretest: 62.7%, C pretest: 55.9%. I posttest: 37.3%, C posttest: 59.3% Between-groups difference: *P* = 0.017 Median (SD) ferritin (ng/mL), posttest: I: 8.7 (0.74), C: 7.9 (0.72), *P* = 0.001 Median (SD)^b^ total iron binding capacity (μg/dl), posttest: I: 540 (13.0), C: 567 (18.8), *P* = 0.001
**Education and consumption monitoring/pill count (iron supplements)**					
Adhikari (2009)^26^	Pill count (unused pills counted in prenatal visits only) for the groups that had pill count as part of the intervention (I1 and I3)	3 months after intervention	Percent of pills taken	Mean (%): I1 posttest: 73% I3 posttest: 88%, *P* < 0.001	Prevalence of anemia (%): I1 pretest: 30%, I1 posttest: 43% I2 pretest: 27%, I2 posttest 19% I3 pretest: 27%, I3 posttest: 18% C pretest: 26%, C posttest: 39% Adjusted odds ratio (95% CI) of anemia prevalence, versus C: I1: 0.93 (0.45, 1.91), *P* > 0.05 I2: 0.41 (0.18, 0.91), *P* < 0.05 I3: 0.35 (0.16, 0.78), *P* < 0.05 Adjusted mean difference (95% CI) of Hb change, versus C: I1: 0.07 (-0.09, 0.23), *P* > 0.05 I2: 0.23 (0.07, 0.39), *P* < 0.01 I3: 0.26 (0.10, 0.42), *P* < 0.01
**Consumption monitoring (iron/iron and folic acid supplements)**					
Ahamed (2018)^28,c^	Pill count I: blister packs collected every week C: blister packs collected every month	100 days after intervention	At least >80% of supplements taken (defined as compliant)	Prevalence compliant (%) I posttest: 69.1 C posttest: 60.4, *P* = 0.001	Mean (SD) Hb: I pretest: 9.38 (0.97), C pretest: 9.49 (1) I posttest: 10.36 (0.98), C posttest: 9.9 (1.15) Mean difference (SD) Hb: I: 0.98 (0.19) (ITT: 0.95 (0.19)), *P* < 0.001 C: 0.46 (0.17), *P* < 0.001 DID: 0.52 g/dL, *P* < 0.001 Prevalence of anemia (%): I pretest: 92.9%, I posttest: 79.9% (ITT: 77.9%), *P* < 0.001 C pretest: 91.8, C posttest: 84.8, *P* < 0.001 DID: 6%, *P* = 0.219
					Mean (SD) ferritin: I pretest: 21.16 (16), I posttest: 34.6 C pretest: 25.2 (22.2), C posttest: 37.3 Mean difference (SD) ferritin: I: 12.8 (6.9) (ITT: 12.6 (6.8)), C: 12.1 (7.1), *P* = 0.198
					Mean (SD) serum transferrin receptor (mg/L): I pretest: 2.5 (1.6), I posttest: 2 C pretest: 2.4 (1.8), C posttest: 1.84 Mean difference (SD): I: −0.5 (−0.33) (ITT: −0.49 (−0.27)), C: −0.56 (−0.37), *P* = 0.873
Balakrishnan (2019)^29^	I: direct observation C: pill count	Once per ANC visit (30–35 days apart for 100 days)	Percent of pills taken (Adherence rate = (no. of pills given - no. of pills remaining) × 100/expected number of pills to be taken)	Mean (SD) adherence rate in % 2nd visit I: 78.48% (10.32), 2nd visit C: 49.22% (15.98), *P* < 0.001 3rd visit I: 79.13% (11.77), 3rd visit C: 52.75% (14.63), *P* < 0.001 4th visit I: 76.44% (9.72), 4th visit C: 53.87% (14.87), *P* < 0.001	Mean (SD) Hb: 1st visit I: 8.97 (1.06), 1st visit C: 8.98 (0.93), *P* = 0.932 2nd visit I: 9.48 (1.07), 2nd visit C: 9.18 (0.99), *P* = 0.130 4th visit I: 9.99 (1.11), 4th visit C: 9.32 (1.04), *P* = 0.006 Prevalence of anemia (%) 1st visit I: 95.8, 1st visit C: 98.5, *P* = 0.653 2nd visit I: 95.2, 2nd visit C: 94.8, *P* = 0.917 4th visit I: 82.8, 4th visit C: 92.6, *P* = 0.116
**SMS reminders (iron supplements)**					
Khorshid (2014)^46^	Pill count	12 weeks after intervention	Number of tablets taken in 3 months (maximum is 84)	Mean (SD) number of iron tablets consumed, posttest: I: 80.5 (10.1), C: 67.2 (21.8), *P* < 0.001 Prevalence (%): High compliance (63–84 tablets), posttest: I: 94%, C: 66%, *P* = 0.03 Moderate compliance (42–62 tablets), posttest: I 4%, C 18% Low compliance (<42 tablets), posttest: I 2%, C 16%	Mean (SD) Hb : I pretest: 11.5 (0.6), I posttest: 11.2 (0.5), *P* < 0.001 pretest: 11.6 (0.6), C posttest: 11.2 (0.9), *P* = 0.002 Between-groups difference: *P* = 0.96 Mean hematocrit (%) (SD): I pretest: 34.4 (1.1), I posttest: 33.9 (1.7), *P* = 0.005 C pretest: 34.9 (1.2), C posttest: 34 (2.6), *P* = 0.01 Between-groups difference: *P* = 0.67
**Multicomponent intervention (iron, folic acid, and calcium supplements)**					
Nguyen (2017)^34^	Recent mothers: self-report Pregnant women: pill count		Number of tablets taken	Mean IFA tablets taken (SD): I pretest: 93.9 (65.9), I posttest 138.6 (45) C pretest: 93.4 (69.2), C posttest 92.1 (63.1), DID: *P* < 0.001 Mean calcium tablets taken (SD): I pretest: 82.5 (63.7), I posttest: 139.6 (44.9) C pretest: 80.7 (67.4), C posttest: 87.7 (63.6), DID: *P* < 0.001	
**Free versus purchased supplements (iron and folic acid supplements)**					
Seck (2009)^41^	Intervention group: pill count Control group: self-report	20 weeks after enrollment	Percent of supplements taken (number of tablets ingested/number of days elapsed since enrollment) × 100	Mean compliance (SD): I posttest: 86.2 (2.4) C posttest: 48.5 (2.9), *P* < 0.001	Mean (SD) Hb: I pretest: 111.8 (1.6), C pretest: 106.5 (1.9), *P* = 0.03 I posttest: 115.9 (2.2), C posttest: 99.8 (2.2), *P* = 0.002 Mean (SD) ferritin: I pretest: 34.1 (3.3), C pretest: 31.7 (2.9), *P* = 0.248 I posttest: 28.7 (2.9), C posttest: 22.0 (2.3), *P* = 0.001 Mean (SD) erythrocyte protoporphyrin concentration (mmol/mol heme): I pretest: 109.1 (7.2), C pretest: 117.8 (10.5), *P* = 0.144 I posttest: 96.5 (8.5), C posttest: 145.3 (9.4), *P* < 0.001
**Participatory action research (iron and folic acid supplements)**					
Shivalli (2015)^31^	Self-report and pill count	12 weeks after intervention	Daily consumption of supplements for at least 100 days (defined as compliant)	Proportion of women compliant (%) I posttest: 85% C posttest: 38% (*P* value for between-groups difference not reported)	Mean (SD) Hb: I pretest: 10.34 (1.56), C pretest: 10.15 (1.59), *P* = 0.577 I posttest: 11.5 (1.24), C posttest: 10.4 (1.38), *P* < 0.001 Prevalence of anemia (%) I pretest: I 65.9%, C pretest: 64%, *P* > 0.05 I posttest: 31.1%, C posttest: 68%, *P* = 0.001
**Capsule versus tablet supplements (iron supplements)**					
Srivastava (2019)^32^	Pill count	Measured monthly, at hospital or home visit by study team, if they didn’t attend hospital visit	At least 90% of supplements taken (defined as good compliance)	Prevalence of good compliance (%): I posttest: 22.0 C posttest: 16.8, *P* > 0.05	Mean (SD) change in Hb, overall: I: 0.79 (1.12), C: 0.44 (1.5), *P* = 0.11, and among good compliants: I: 1.19 (1.01), C: 0.83 (1.37), *P* = 0.41 Mean (SD) change ferritin, overall: I: −0.8 (19.2), C: −1.14 (30.8), *P* = 0.93, and among good compliants: I: 2.50 (20.18), C: −2.14 (6.93), *P* = 0.42

Note: When available, outcome results are highlighted in green if differences (within-group and between-groups) are statistically significant, and in red when not statistically significant.

a These are the results as reported by the study authors, but they are likely to have been expressed as SD (mean), rather than mean (SD).

b Authors recognized that the data are not normally distributed and presented them as median (SD).

c The study from Ahamed 2018 was the only study (of the randomized controlled trials) reporting side effects. Side effects were expressed as prevalence (% of women) experiencing: (1) nausea/vomiting: I posttest: 57.6%, C posttest: 36.5%; (2) upper abdominal pain: I posttest: 38.5%, C posttest: 17.4%; (3) constipation/diarrhea: I posttest: 7.7%, C posttest: 9.5%.

C, control group; I, intervention group; DID, difference-in-difference ((CI), *P*-value); Hb, hemoglobin; IFA, iron and folic acid supplements; ANC, antenatal care.

**Table 4.** Risk of bias summary for nonrandomized studies (ROBINS-I tool)

Study author (year)	Bias due to confounding	Bias in selection of participants into the study	Bias in classification of intervention	Bias due to deviations from intended intervention	Bias due to missing data		Bias in measurement outcomes		Bias in selection of the reported result	Overall bias
					Pregnancy and birth outcomes	Adherence	Pregnancy and birth outcomes	Adherence		
Anitasari (2017)^35^	Moderate	Low	Moderate	Low	Low	Moderate	Low	Moderate	Low	Moderate
Chaiopanont (2019)^40^	Low	Moderate	Low	Low	Moderate	Low	Low	Serious	Moderate	Serious
Darmayanti (2019)^36^	No information	No information	Low	No information	No information	Low	No information	No information	Low	No information
Hazra (2019)^33^	Moderate	Moderate	Low	Moderate	Not applicable	Low	Not applicable	Moderate	Low	Moderate
Kamau (2020)^42^	Low	Serious	Low	Serious	Not applicable	Low	Not applicable	Moderate	Low	Serious
Prinja (2017)^27^	Serious	Low	No information	Low	Low	Low	Serious	Serious	Low	Serious
Surtimanah (2019)^25^	Critical	No information	Low	No information	Not applicable	Low	Not applicable	Low	Low	Critical
Vernissa (2017)^37^	Serious	Low	Low	No information	Serious	Low	Moderate	Moderate	Low	Serious

**Table 5.** Risk of bias summary for randomized controlled trials (Cochrane risk-of-bias tool)

Study author (year)	Sequence generation	Allocation concealment	Blinding of participants and personnel		Blinding of outcome assessors		Incomplete outcome data		Selective outcome reporting	Other sources of bias
			Pregnancy and birth outcomes	Adherence	Pregnancy and birth outcomes	Adherence	Pregnancy and birth outcomes	Adherence		
Abujilban (2019)^43^	Low	Low	Low	High	Low	Unclear	Low	Low	Low	Low
Adhikari (2009)^26^	Low	Low	Low	High	Low	Low	Low	Low	Low	Low
Ahamed (2018)^28^	Low	High	Low	High	Low	Low	Low	Unclear	Low	Low
Balakrishnan (2019)^29^	Low	Unclear	Low	High	Low	High	Low	Low	Low	Low
Heryadi (2017)^38^	Unclear	Unclear	Low	Unclear	Low	Unclear	Low	Low	Low	Unclear
Jalambadani (2018)^44^	Low	Unclear	Low	Unclear	Low	Unclear	Unclear	Unclear	Low	Unclear
Jalambadani (2020)^45^	Unclear	Unclear	Low	Unclear	Unclear	Unclear	Unclear	Unclear	Low	Unclear
Khorshid (2014)^46^	Low	Unclear	Low	High	Low	Unclear	Low	Low	Low	Low
Nahrisah (2020)^39^	Low	Low	Low	Low	Low	Unclear	Low	Low	Low	Low
Nguyen (2017)^34^	Low	Low	Not applicable	Unclear	Not applicable	Unclear	Not applicable	Low	Low	Low
Saha (2018)^30^	Unclear	Unclear	High	High	Low	Unclear	High	High	Low	Unclear
Seck (2009)^41^	Low	High	Low	Unclear	Low	Unclear	Low	Low	Low	Low
Shivalli (2015)^31^	Unclear	Unclear	Low	High	Low	High	Low	Low	Low	Low
Srivastava (2019)^32^	Low	Low	Low	Unclear	Low	Low	Low	Low	Low	Low

## Footnotes

a mHealth, as an abbreviation of mobile health, is a term used for the practice of medicine and public health supported by mobile devices. In this study, the mHealth application served as a job aid for community health volunteers to provide customized counseling messages for pregnant women, using economical, Java-based mobile phones. The application had tailored content with locally relevant audio and visual prompts to equip community health volunteers with multimedia job aids to support client assessment, counseling, early identification, treatment, and/or rapid referral of pregnancy, postpartum, and newborn complications.
