# Supplementary material for: Interventions to increase adherence to micronutrient supplementation during pregnancy: a systematic review
Source: Ann N Y Acad Sci. 2021 Jan 5;1493(1):41–58. doi: 10.1111/nyas.14545 (PMC8169578; doi:10.1111/nyas.14545)
Supplement: Supplementary file 1 — Supplementary Material [file NYAS-1493-41-s001.docx]

Search strategy used in MEDLINE (via Pubmed)

1. Search ((Pregnancy[MeSH Terms]) OR (Pregnant Women[MeSH Terms]) OR (Prenatal Care[MeSH Terms]) OR(pregnan*[Title/Abstract]) OR (gestat*[Title/Abstract]) OR (perinatal[Title/Abstract]) OR (parturi*[Title/Abstract]))

2. Search ((micronutrients[MeSH Terms]) OR (minerals[MeSH Terms]) OR (24,25-dihydroxyvitamin[Title/Abstract]) OR (D3[Title/Abstract]) OR (25-hydroxyvitamin[Title/Abstract]) OR (D2[Title/Abstract]) OR (acetylcarnitine [Title/Abstract]) OR (alpha-tocopherol*[Title/Abstract]) OR ("4-aminobenzoic acid*"[Title/Abstract]) OR ("aminobenzoic acid*"[Title/Abstract]) OR ("ascorbic acid*"[Title/Abstract]) OR ("beta carotene"[Title/Abstract]) OR (beta-tocopherol*[Title/Abstract]) OR (biotin [Title/Abstract]) OR (boron [Title/Abstract]) OR (cadmium [Title/Abstract]) OR (calcifediol*[Title/Abstract]) OR (calcitriol*[Title/Abstract]) OR (calcium [Title/Abstract]) OR (carnitine [Title/Abstract]) OR (caroten*[Title/Abstract]) OR (cholecalciferol*[Title/Abstract]) OR (chromium [Title/Abstract]) OR (cobalt [Title/Abstract]) OR (cobamide*[Title/Abstract]) OR ("cod liver oil"[Title/Abstract]) OR (copper [Title/Abstract]) OR ("dehydroascorbic acid*"[Title/Abstract]) OR (dihydrotachysterol*[Title/Abstract]) OR (dihydroxycholecalciferol*[Title/Abstract]) OR (ergocalciferol*[Title/Abstract]) OR (Flavin [Title/Abstract]) OR (folate [Title/Abstract]) OR ("folic acid*"[Title/Abstract]) OR (formyltetrahydrofolate*[Title/Abstract]) OR (fursultiamin [Title/Abstract]) OR (gamma-tocopherol*[Title/Abstract]) OR (hydroxocobalamin [Title/Abstract]) OR (hydroxycholecalciferol*[Title/Abstract]) OR (inositol [Title/Abstract]) OR (iodine [Title/Abstract]) OR (iron[Title/Abstract]) OR (leucovorin [Title/Abstract]) OR (magnesium [Title/Abstract]) OR (manganese [Title/Abstract]) OR (micronutrient*[Title/Abstract]) OR (mineral*[Title/Abstract]) OR (molybdenum [Title/Abstract]) OR (mononucleotide*[Title/Abstract]) OR (niacin [Title/Abstract]) OR (niacinamide [Title/Abstract]) OR (nickel [Title/Abstract]) OR (nicorandil [Title/Abstract]) OR ("nicotinic acid*"[Title/Abstract]) OR (palmitoylcarnitine [Title/Abstract]) OR ("pantothenic acid*"[Title/Abstract]) OR ("pteroylpolyglutamic acid*"[Title/Abstract]) OR (pyridoxal [Title/Abstract]) OR ("pyridoxal phosphate"[Title/Abstract]) OR (pyridoxamine [Title/Abstract]) OR (pyridoxine [Title/Abstract]) OR (riboflavin [Title/Abstract]) OR (selenium [Title/Abstract]) OR (silicon [Title/Abstract]) OR (tetrahydrofolate*[Title/Abstract]) OR (thiamine [Title/Abstract]) OR ("thioctic acid*"[Title/Abstract]) OR (tin [Title/Abstract]) OR (tocopherol*[Title/Abstract]) OR (tocotrienol*[Title/Abstract]) OR ("trace element*"[Title/Abstract]) OR (ubiquinone[Title/Abstract]) OR (vanadium[Title/Abstract]) OR (vitamin*[Title/Abstract]) OR (zinc[Title/Abstract]))

3. Search ((Dietary Supplements[MeSH Terms]) OR (Administration, Oral[MeSH Terms]) OR (Capsules[MeSH Terms]) OR (Tablets[MeSH Terms]) OR (Powders[MeSH Terms]) OR (supplement*[Title/Abstract]) OR (pill*[Title/Abstract]) OR (capsule*[Title/Abstract]) OR (tablet*[Title/Abstract]) OR (liquid*[Title/Abstract]) OR (oral*[Title/Abstract]) OR (dietary supplements[Title/Abstract]))

4. Search ((Treatment Adherence and Compliance[MeSH Terms]) OR (Adherence[Title/Abstract]) OR (Compliance[Title/Abstract]) OR (Uptake[Title/Abstract]) OR (Consumption[Title/Abstract]))

5. Search ((#1) AND #2) AND #3) AND #4))
